# Optimized Land–Water Management Expands Afforestation Potential Under Land and Human Water‐Use Constraints

**DOI:** 10.1002/advs.77990

**Published:** 2026-10-11

**Authors:** Binbin Huang, Fei Lu, Yuebo Su, Xuming Wang, Hua Zheng, Zhiyun Ouyang

**Affiliations:** ^1^ State Key Laboratory of Urban and Regional Ecology Research Center for Eco‐Environmental Sciences Chinese Academy of Sciences Beijing China; ^2^ University of Chinese Academy of Sciences Beijing China; ^3^ Beijing‐Tianjin‐Hebei Urban Megaregion National Observation and Research Station for Eco‐Environmental Change Beijing China; ^4^ Yunnan Key Laboratory of Forest Ecosystem Stability and Global Change Xishuangbanna Tropical Botanical Garden Chinese Academy of Sciences Kunming Yunnan China; ^5^ Key Laboratory for Humid Subtropical Eco‐Geographical Processes of the Ministry of Education College of Geographical Sciences Fujian Normal University Fuzhou China

**Keywords:** afforestation, carbon sink, climate change, human water demand, land constraints, optimized land management, optimized water management

## Abstract

Afforestation is a low‐cost and high‐potential climate change mitigation strategy, but its feasibility remains highly uncertain because most existing assessments focus primarily on biophysical suitability and land resource constraints, while largely overlooking the critical limitation imposed by human water demand. Here, we developed an integrated framework combining machine learning, statistical modelling, the Biome‐BGCMuSo model, and multi‐scenario analysis to quantify China's afforestation potential under coupled land and water constraints and explore how optimized land–water management can enhance afforestation potential and associated carbon sink. We found human water demand reduced China's feasible afforestation potential from 78.13 to 40.57 Mha, with climate change causing a further 15.23%–15.91% decline. Notably, optimized land management and water‐saving measures could individually increase feasible afforestation area by 6.04–7.05 Mha and 5.89–6.97 Mha, respectively. When combined, these measures would further increase afforestation potential by 14.95 and 13.66 Mha under SSP‐245 and SSP‐585, respectively, generating an additional carbon sink of 109.64 ± 12.08 and 125.92 ± 13.40 Tg C yr^−^^1^ by 2060. Our findings reveal that ignoring human water demand substantially overestimates afforestation potential and identify practical pathways to expand feasible afforestation potential while minimizing land‐use conflicts and safeguarding water security under future climate change.

## Graphical abstract

Human water demand constrains China's feasible afforestation potential more strongly than is commonly recognized, and future climate change further intensifies this limitation. Integrating land‐use optimization with water‐saving measures substantially expands feasible afforestation area and associated carbon sinks, revealing a practical pathway to reconcile climate mitigation, food production, and water security.

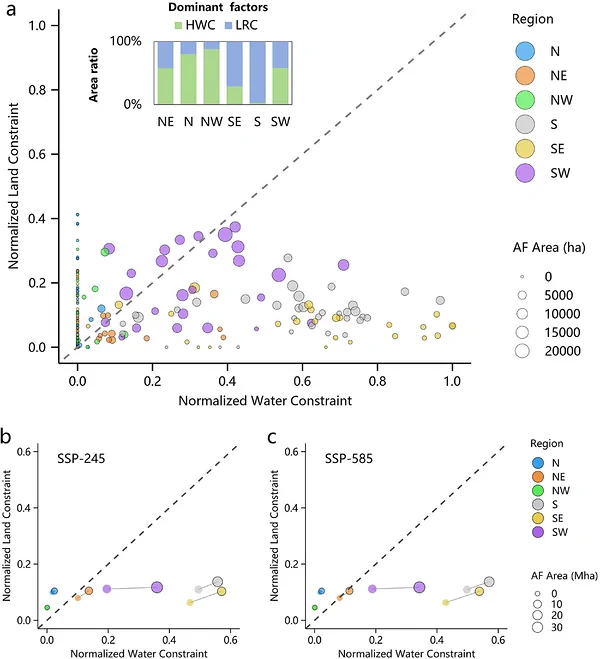

## 1 Introduction

Afforestation programs have been the dominant driver of increases in China's terrestrial carbon sink over the past few decades [1, 2]. Prior studies have projected that China's forest carbon sink could be doubled through continued afforestation in the future [3, 4]. However, these estimates focus predominantly on land resource constraints, and overlooking the intensifying conflict between growing socioeconomic water demands and the water consumption required for tree planting and forest growth, particularly in water‐stressed regions [5]. This neglecting probably caused the overestimate of China's afforestation potential.

The constraints imposed by land resource suitability on afforestation potential have been widely estimated in prior studies [3, 4, 6, 7]. The constraints of land resources include not only biophysical suitability (e.g., soil, climate, and terrain compatibility) but also intense competition with other land use demands, including those related to agriculture, human settlements, and urban development [8, 9]. Consequently, not all areas that are biophysically suitable for afforestation can be converted to forest, as many are already allocated to — or prioritized for — food production, residential use or urbanization. Prior study implied that the global tree restoration potential would decline from 1.6 billion ha to 0.9 billion ha after excluding the land used for human development and agriculture [6]. Cropland has historically been the main source of new forests in China (1990–2020) [10, 11], but with the heightened national priority given to food security, further large‐scale conversion of arable land is becoming increasingly restricted [12].

In addition to land resource suitability, human water needs impose a significant constraint on the potential for large‐scale afforestation. The exhausted water through afforestation has been proven to reach the limits of Loess Plateau's water carrying capacity, and continued large‐scale planting could pose substantial risks to water security for local livelihoods and production systems [13]. Achieving the full global potential for ecological restoration would decrease per capita water availability by ∼26%, but safeguarding human water use would diminish the associated carbon sink potential by 1/3 [5]. Notably, China ranks as the world's largest freshwater withdrawing nation, supporting nearly 20% of the global population and generating more than 18% of the global GDP, with only 6%–7% of the world's renewable freshwater resources [14]. China also faces acute water scarcity, with approximately one‐third of its provinces exhibiting per capita water availability below the internationally recognized threshold for severe scarcity [15]. Projections indicate that China's future water consumption will continue to increase under combined socioeconomic development and climate change scenarios, potentially exacerbating water stress [16, 17]. Consequently, the constraining effect of human water consumption on afforestation potential is poised to become increasingly acute in the coming decades.

Notably, constraints from land resources and human water demand have potential to be alleviated through optimized land and water management. China's rural land use efficiency remains low because of severe farmland fragmentation, rapid urbanization, rural depopulation, and widespread abandonment of homesteads and fallow fields [18, 19]. Optimizing these underutilized lands through consolidation, reclamation, and transfer could release substantial areas for afforestation while maintaining or even enhancing agricultural output. Similarly, water use efficiency in China's production and domestic sectors remains relatively low, with water consumption per unit of GDP remaining significantly higher than the levels observed in developed countries, highlighting a substantial efficiency gap [15]. If the Chinese government implements measures to promote water use efficiency in the future, the water surplus suitable for afforestation will likely further improve in the future.

Currently, China's water consumption was by approximately 519.37 × 10^9^ m^3^, the human water demand overpasses the water demand occurred in over half of the total basins (Figure 1). Owing to economic growth and climate change, this magnitude is projected to increase by 25.58% (SSP‐245) and 52.57% (SSP‐585) in 2060, respectively (Figure S1). Thus, it is necessary to optimize the management of land and water resources to relieve their constraints on future afforestation. To explore the potential improvement in carbon sinks resulting from China's afforestation under optimized management, we integrated machine learning, statistical modelling, process‐based simulations, and multi‐scenario analysis (including scenarios involving different land and water constraints and optimized management strategies; further details are provided in the Experimental Section). First, we quantified the effects of land resource constraints and water security limitations on China's feasible afforestation potential and associated carbon sink; then, we explored the extent to which optimized land and water management strategies can enhance future afforestation potential and amplify carbon sink contributions.

**FIGURE 1:**
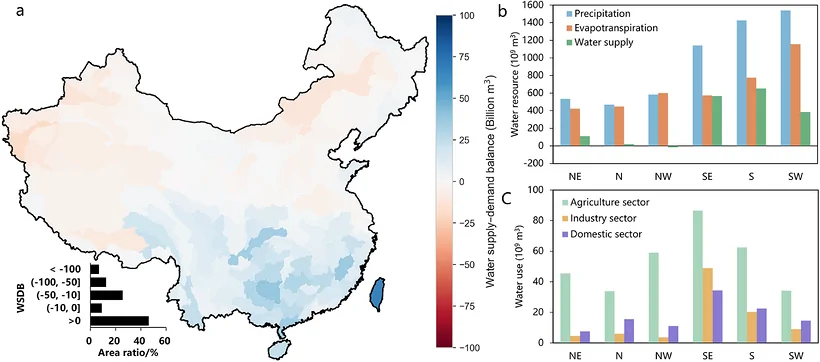
The spatial pattern and magnitude of water supply–demand balance in 2020. (a) the spatial pattern and magnitude of water supply–demand balance; (b) the magnitude of precipitation‐evapotranspiration‐water supply in different China's sub‐regions; (c) the magnitude of water uses in agriculture, industry and domestic sectors in different China's sub‐regions. WSDB, water supply–demand balance; N, northern China; NE, northeastern China; NW, northwestern China; S, southern China; SE, southeastern China; SW, southwestern China.

## 2 Results

If we consider only the effect of land resource constraints, an area of approximately 78.13 Mha is suitable for afforestation (Figure 2, Figure S2), with a potential carbon sink of 246.83 ± 27.32 TgC yr^−1^ in 2060 (Figure 2, Figure S3). However, when the constraints of human water demand are considered, the water resource supply cannot meet the human water needs in more than half of China's total basins (Figure 1), these areas are no longer suitable for afforestation. Given our assumption, if we planted new trees only in basins where the water supply need did not surpass human water needs, the potential areas suitable for afforestation would decline to 40.57 Mha (Figure 2). The carbon sink potential contributed by these new planted forests would thus decrease to 167.95 ± 18.32 TgC yr^−1^ (Figure 2, Figure S3). Moreover, the gap between water supply and human water demand would enlarge owing to the economic growth and climate change (Figure S1). The change would further decrease the potential afforestation area to 34.15 Mha (SSP‐245) and 34.42 Mha (SSP‐585), and the carbon sink further decrease to 68.57 ± 8.90 Tg C yr^−^^1^ (SSP‐245) and 82.08 ± 10.56 Tg C yr^−^^1^ (SSP‐585) in 2060, respectively (Figure 2).

**FIGURE 2:**
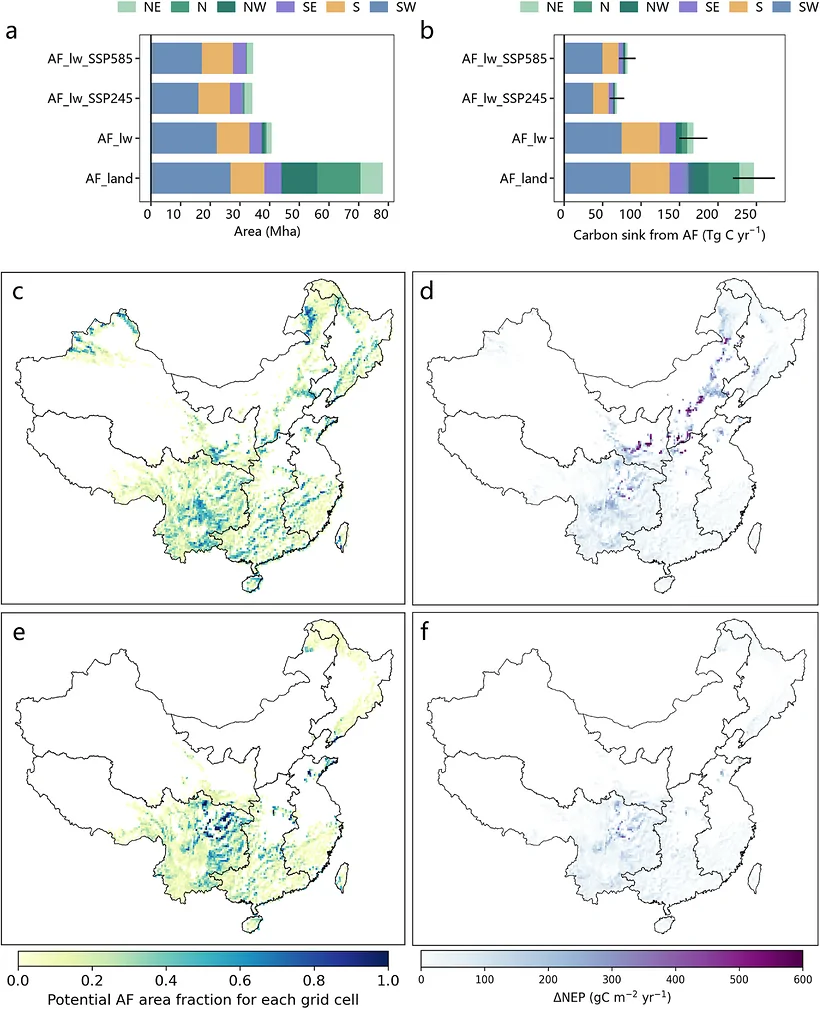
Afforestation potential and associated carbon sink. (a) the magnitude of potential afforestation area under the constraint of land resources and human water demand; (b) carbon sink from potential afforestation under the constraint of land resources and human water demand; spatial pattern of (c) potential afforestation area and (d) corresponding carbon sink under constraint of land resources only; spatial pattern of (e) potential afforestation area and (f) corresponding carbon sink under constraint of land resources and human water demand. AF, afforestation; AF_lw, current afforestation potential under land‐resource and human water‐demand constraints; AF_lw_SSP245, afforestation potential under land‐resource and human water‐demand constraints in 2060 under SSP2–4.5; AF_lw_SSP585, afforestation potential under land‐resource and human water‐demand constraints in 2060 under SSP5–8.5; N, northern China; NE, northeastern China; NW, northwestern China; S, southern China; SE, southeastern China; SW, southwestern China.

Given that the policy of cropland occupation–compensation balance constrains land availability for afforestation, we proposed optimized land management strategy. This strategy aims to expand cropland in plains via land‐use rights transfer, land consolidation, and reclamation of abandoned cropland, while shifting afforestation to areas with steeper slopes and less favorable cultivation conditions at the same area (Figure 3, Figure S4). It would contribute 15.60 Mha and 15.65 Mha to support afforestation under the SSP‐245 and SSP‐585 scenarios (Figure 3). But when we consider the constraints of human water demand, the increased afforestation potential from optimized land management would decline to 45.21% (7.05 Mha, SSP‐245) and 41.17% (6.49 Mha, SSP‐585), leading carbon sinks from afforestation reach to 80.23 ± 10.50 Tg C yr^−^^1^ (SSP‐245) and 92.16 ± 11.35 Tg C yr^−^^1^ (SSP‐585) in 2060, respectively (Figures 3 and 4, Figure S5).

**FIGURE 3:**
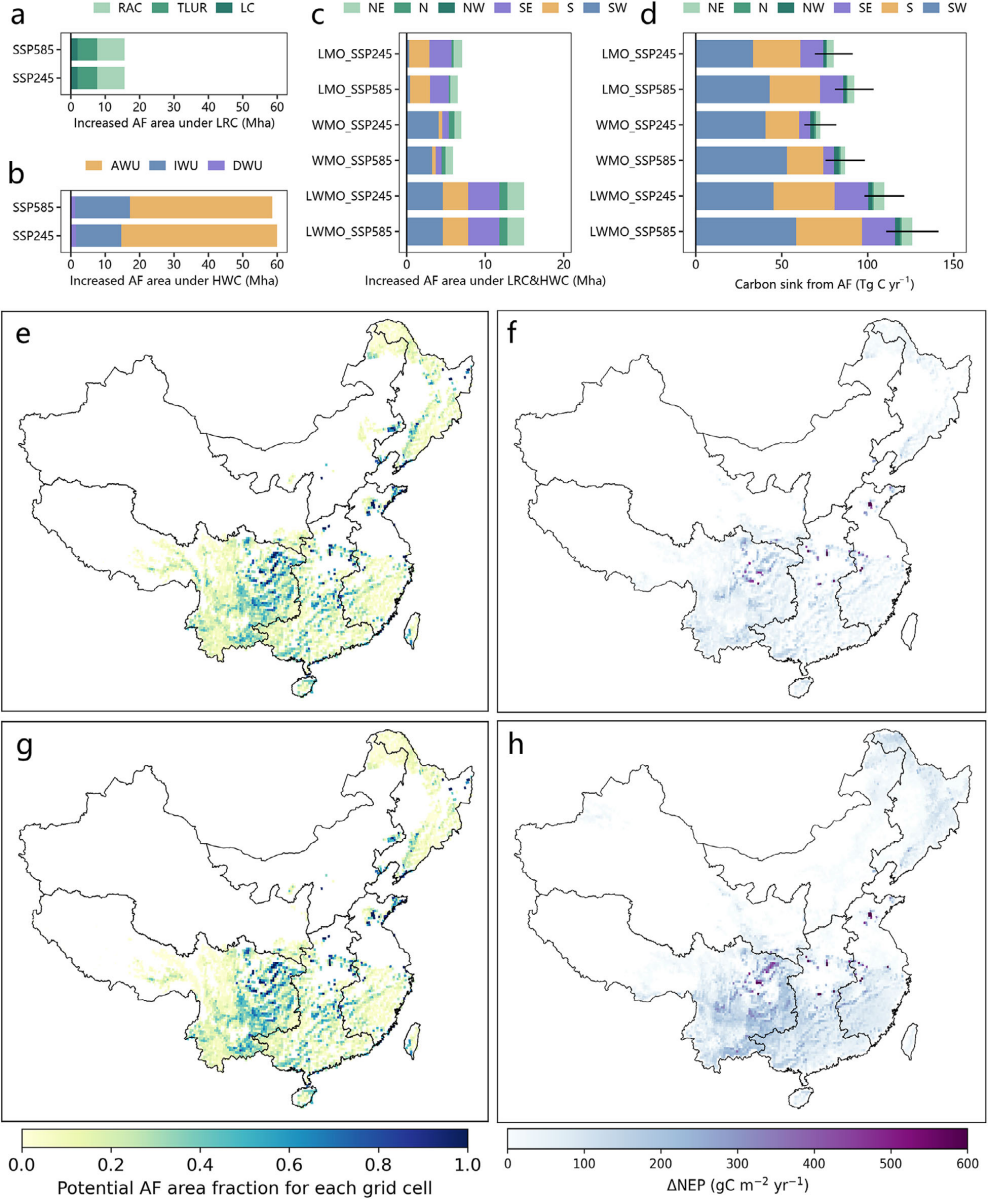
Increased afforestation potential and associated carbon sink under optimized land management and water management. (a) the magnitude of increased afforestation potential through optimized land management under LRC; (b) the magnitude of increased afforestation potential through optimized water management under HWC; the magnitude of (c) increased afforestation potential and (d) corresponding carbon sink through different optimized management scenarios under LRC and HWC; spatial pattern of (e) afforestation potential from LWMO and (f) corresponding carbon sink under LRC and HWC under SSP‐245 scenario in 2060; spatial pattern of (g) afforestation potential from LWMO and (h) corresponding carbon sink under LRC and HWC under SSP‐585 scenario in 2060. LRC, land‐resource constraint; HWC, human water‐demand constraint; RAC, reclamation of abandoned cropland; TLUR, transfer of land‐use rights; LC, land consolidation; AWU, agricultural water use; IWU, industrial water use; DWU, domestic water use; LMO, land‐management optimization scenario; WMO, water‐management optimization scenario; LWMO, land–water management optimization scenario; **ΔNEP**, change in net ecosystem productivity; N, northern China; NE, northeastern China; NW, northwestern China; S, southern China; SE, southeastern China; SW, southwestern China.

**FIGURE 4:**
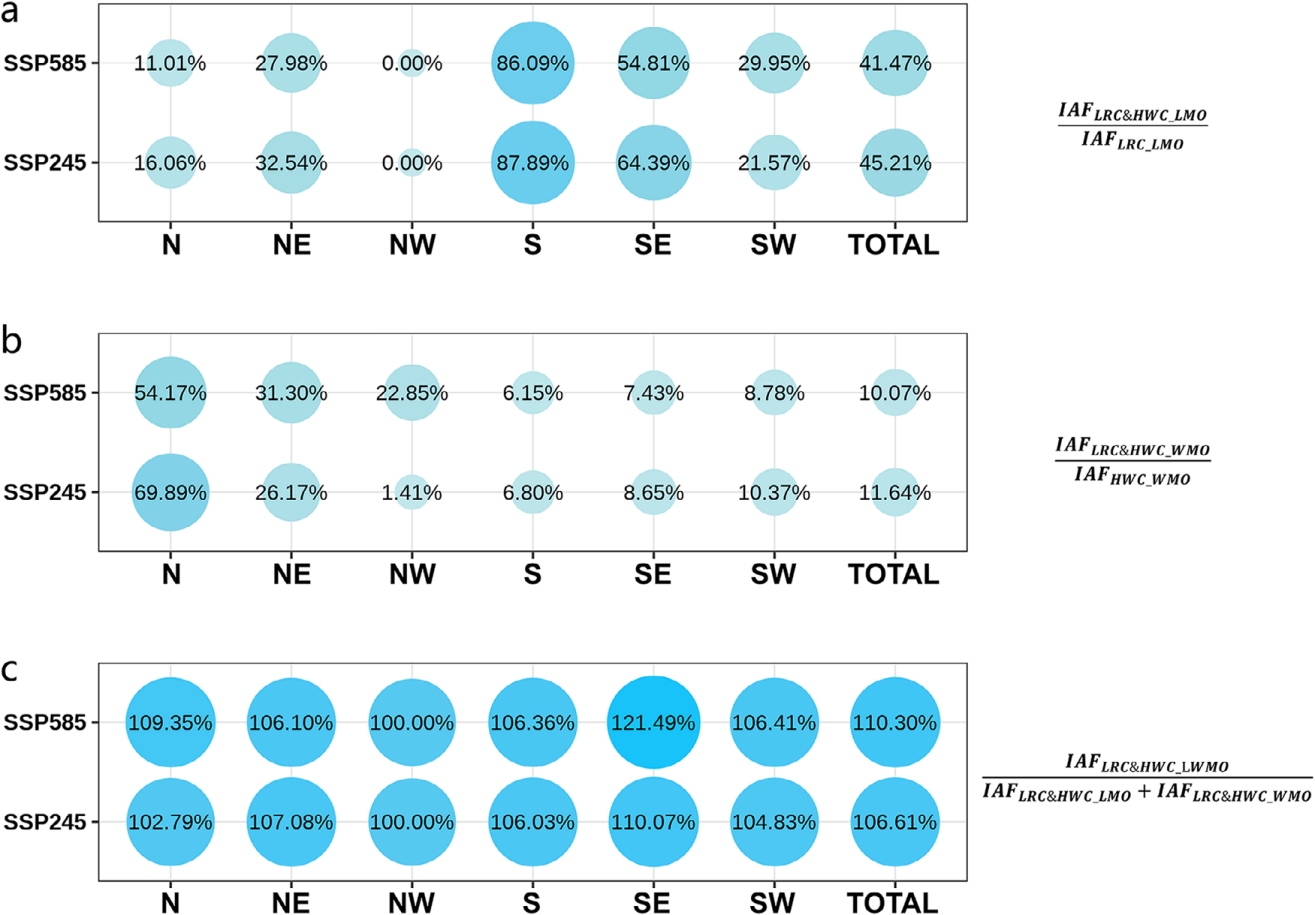
The efficiency rate of land and water management on increasing afforestation potential. (a) the efficiency rate of IAF from LMO under constraints of LRC&HWC to IAF from LMO under constraints of LRC alone; (b) the efficiency rate of IAF from WMO under constraints of LRC&HWC to IAF from WMO under constraints of HWC alone; (c) the efficiency rate of IAF from LWMO under constraints of LRC&HWC to the sum of IAF from LMO under constraints of LRC&HWC and IAF from WMO under constraints of LRC&HWC. IAF, increased afforestation potential; LRC, land‐resource constraint; HWC, human water‐demand constraint; LMO, land‐management optimization scenario; WMO, water‐management optimization scenario; LWMO, land–water management optimization scenario; N, northern China; NE, northeastern China; NW, northwestern China; S, southern China; SE, southeastern China; SW, southwestern China.

Similarly, optimized water management strategy can also increase water surplus through water‐saving measures in the agricultural, industrial, and domestic sectors to support afforestation (Figures S1, S6, and S7. Our results revealed optimized water management could reduce water consumption by 36.08% (SSP‐245) and 39.89% (SSP‐585) compared with the BAU scenario in 2060 (Figure S1). Compared with the current water consumption, the rates of decrease would further reach 45.22% (SSP‐245) and 55.26% (SSP‐585), respectively (Figures S1, S6, and S7). The saved water from optimized water management is expected to support afforestation by 58.59 Mha (SSP‐245) and 59.92 Mha (SSP‐585) additionally (Figure 3). But when the land resource constraints is considered, the increased feasible afforestation potential from optimized water management would decline to 11.64% (6.97 Mha, SSP‐245) and 10.07% (5.90 Mha, SSP‐585), leading carbon sinks from afforestation reach to 72.42 ± 9.28 Tg C yr^−^^1^ (SSP‐245) and 86.87 ± 9.02 Tg C yr^−^^1^ (SSP‐585) in 2060, respectively (Figures 3 and 4, Figure S8).

Furthermore, when optimized land and water management are implemented together, the feasible afforestation potential increases by 14.95 Mha (SSP‐245) and 13.66 Mha (SSP‐585), which are greater than the sum of the contributions of the individual strategies and leading carbon sinks from afforestation further reach to 109.64 ± 12.08 Tg C yr^−^^1^ (SSP‐245) and 125.92 ± 13.40 Tg C yr^−^^1^ (SSP‐585) in 2060, respectively (Figures 3 and 4).

## 3 Discussion

### 3.1 Constraints of Human Water Needs on Afforestation and the Associated Carbon Sink

Prior studies have estimated China's afforestation and its contribution to carbon sinks, they have concluded that China's future afforestation potential was approximately 69.95 ± 8.51 Mha [3, 4, 20, 21]. This magnitude is consistent with the result of our estimation (78.13 Mha) considering only land resource constraints (Figure 2). Such discrepancies mainly arise from using different land use classification product, and the treatment of competing land demands and the biophysical and policy constraints considered [22, 23]. Previous studies have indicated that drought can reduce the tree survival rate in northern China and cause forests to die, aggravating CO_2_ emissions [24, 25]. If human water demand is accounted for, the afforestation potential in arid regions will further decrease, as the water supply in many northern basins already falls short of the human water demand (Figure S9). Moreover, future climate change, socioeconomic development and population dynamics are projected to increase total water consumption by 44.82%–62.69% (Figure S1). Consequently, the gap between water supply and demand is expected to widen (Figure S1), even if total water resources increase under future climate conditions (Figure S6). Notably, China's domestic water use was projected to exhibit an initial increase followed by a decline (Figure S1). The main cause was the urban residents have higher per‐capita domestic water‐use rates than rural residents (Figure S10), the initial growth in the urban population offset the decrease in water use caused by the declining rural population, thereby driving a further increase in domestic water use. However, as the urban population also began to decline in later years (Figure S11), total domestic water uses eventually decreased. Nevertheless, the domestic water use in 2060 is still higher than that in 2020 (Figure S1). Although the afforestation potential was estimated at 78.13 Mha under land‐resource constraints alone and at 164 Mha under human‐water‐demand constraints alone, it decreased substantially to 40.57 Mha when both constraints were considered simultaneously (Figure 2 and Figure S12). This reduction was mainly attributable to the spatial mismatch between land‐resource constraints and human‐water‐demand constraints (Figure S9). Human water demand was the main constraint on afforestation potential in northwestern and northern China, whereas land resources were the main constraint in southern, southeastern and southwestern China (Figure 5). Compared with the former area, the latter areas are the primary agricultural production zones. They are characterized predominantly by hilly and mountainous terrain, which results in limited land availability, generally low land use efficiency, and widespread farmland fragmentation [19]. Highly fragmented and small land parcels constrain large‐scale farming practices, thereby limiting improvements in crop yields. In addition, farmland fragmentation reduces the efficiency of input use (e.g., fertilizers), increases the risk of nonpoint source pollution, and ultimately suppresses agricultural productivity [18].

**FIGURE 5:**
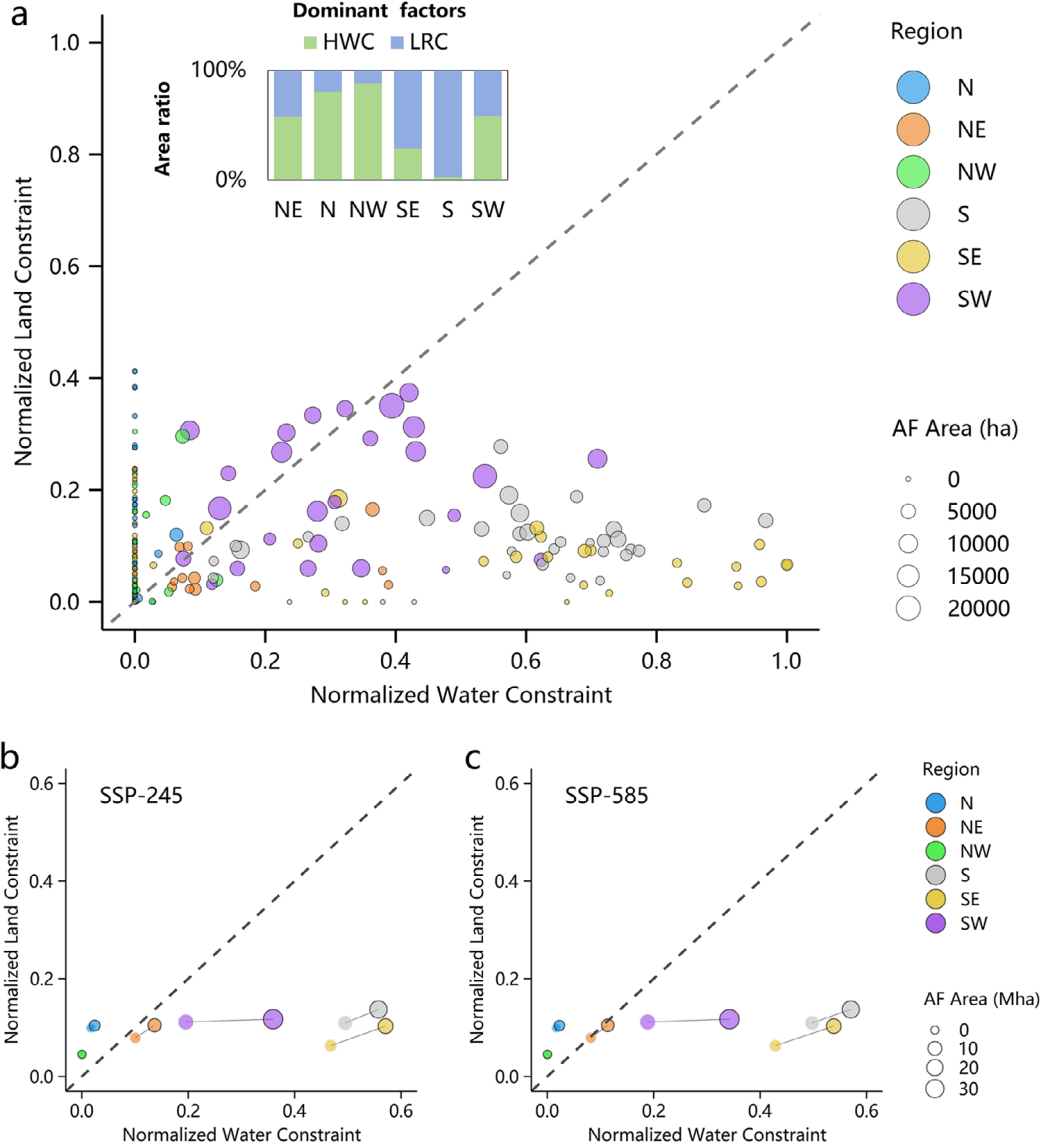
Normalized constraint from land and water on afforestation potential by basin and region. (a) Normalized constraint from land and water on afforestation potential by basin; Normalized constraint from land and water on afforestation potential by (b) region under SSP‐245 in 2060 and (c) region under SSP‐585 in 2060. In sub‐figure b and c, bubbles with grey borders represent the pre‐optimization scenario, while those with black borders represent the post‐optimization (optimized land management and water management) scenario. The bar plot denotes the area ratio belongs to different dominant factors across different sub‐regions. Normalized Land Constraint is defined as the ratio of land‐supported afforestation potential to the total regional area, while Normalized Water Constraint is defined as the ratio of water‐supported afforestation potential to the total regional area. AF, Afforestation; LRC, land‐resource constraint; HWC, human water‐demand constraint; N, northern China; NE, northeastern China; NW, northwestern China; S, southern China; SE, southeastern China; SW, southwestern China.

### 3.2 Effects of Optimized Land and Water Management on Afforestation and Its Carbon Sink

Optimized land management can relieve the constraints of land resources on afforestation potential, but the magnitude would largely decrease after the constraints of human water demand are considered (Figure 3). Moreover, the proportion varied across the different subregions. It decreased even further to zero in Northwest China, while in South China, it could exceed 80% (Figure 4). The variation in water surplus to support afforestation in different subregions is the main reason for this variation (Figure S10). These findings further suggest that improving water use efficiency to reduce water consumption or alleviating water scarcity through inter‐basin water transfer project [26] could unlock greater afforestation potential from land management optimizing.

Optimized land management is not only helpful for increasing afforestation potential but can also reduce cropland fragmentation, which could further increase grain production through the adoption of mechanized farming. Currently, more than 70% of China's farmland patches have areas smaller than 0.6 ha, and more than 90% are smaller than 16 ha [19], these farmland patches contribute only 8% of the national total yield production [18]. Although the rural population is 20% lower than the urban population, rural residential and built‐up areas are 160% larger [27]. Rapid urbanization has driven massive young rural labourers to migrate to cities, resulting in the widespread idleness and abandonment of rural land. These circumstances suggest the substantial waste of China's already scarce land resources. Additionally, rapid urbanization has driven China's farmland to shift from flat eastern regions to steeper slopes in the northwest, resulting in a sharp increase in agricultural water consumption [28]. Optimizing land management can effectively increase the area of high‐quality cropland, enabling the conversion of more low‐quality sloping cropland into forests while ensuring that the total cropland area does not decrease and crop yield does not decline. This achieves a win–win outcome for both food security and climate change mitigation. Additionally, a prior study suggested that optimizing land management can reduce environmental pollution by preventing the overuse of chemical fertilizers and pesticides [29].

Similarly, although water‐saving strategies could increase the water surplus‐supported afforestation area by 58.59–59.92 Mha (Figure 3), the feasible afforestation potential decreased to 10.07%–11.64% after the constraints of land resources are considered (Figure 4). Different from optimized land management, this efficiency ratio of increased afforestation potential is much greater in North China than South China under optimized water management (Figures 4 and 5). Spatial heterogeneity in the dominant limiting factors of afforestation potential is the primary reason for this difference (Figure 5, Figure S9).

The combined implementation of optimized land management and water‐saving strategies produced synergistic effects, jointly alleviating land resource and human water demand constraints and thereby enabling greater afforestation potential (Figure 3). Furthermore, optimized management can mitigate the risk of crop yield losses caused by water shortages [30] and reduce greenhouse gas emissions associated with agricultural and industrial production processes, indirectly contributing to carbon neutrality goals [31, 32, 33]. Notably, the southern area would obtain greater improvement in afforestation potential than that in north area suggest the constraints of human water demand are greater than land resources (Figure 5).

### 3.3 Implications

This study demonstrated that China's afforestation potential was largely overestimated previously because only the constraints of land resources without human water demand were considered. Moreover, our results indicate that optimized land management and water management can largely mitigate these constraints. Globally, many regions—especially in developing countries—show a sharp decline in ecological restoration potential once human water demands are considered [5]. Like China, many developing countries in South America, Africa, and Southeast Asia also face low efficiency in terms of land and water use [34, 35, 36]. If optimized land and water management are implemented in these regions, the constraints imposed by human water consumption would largely be alleviated, yielding greater potential for afforestation and the resulting increase in the carbon sink towards the achievement of climate mitigation goals. Moreover, such efforts could support the realization of other sustainable development goals [37].

### 3.4 Uncertainty Analysis

Previous global‐scale studies have shown that China's achievable carbon sequestration potential under water constraints is only 16.7% [5], which is much lower than our results. The discrepancies arise from several factors. (1) Previous studies estimated the potential carbon storage associated with restoration, whereas our study quantifies the carbon sink potential only from afforestation. (2) Earlier estimates were based on the full extent of potential natural vegetation (PNV), whereas our analysis restricts afforestation to areas excluding impervious surfaces, wetlands, and croplands; (3) Prior research was conducted at the national scale, whereas our study is based on the basin scale. Large countries often span multiple climate zones, where water supply and demand can vary considerably across space. Compared with global‐scale assessments, which uses countries as the minimum unit for water supply–demand balance, we focus on China and uses basins as the minimum unit. At this scale, water supply, demand, and hydrological processes are more clearly represented. Basin scale can more accurately captures the spatial heterogeneity of the constraints imposed by human water demand on afforestation potential in China, and reducing uncertainties arising from large‐scale mismatches between water supply and demand. Meanwhile, here we used the threshold of tree cover≥20% rather than 10% (adopted by FAO) to define forests. This threshold is consistent with the forest definition used in China's National Forest Inventory [38] and previous studies that estimated forest extent and afforestation potential in China [3, 4, 39], allowing for better comparison with the findings of those studies.

Demographic shifts is an important factor to determining the change of domestic water use. However, demographic shifts indirectly affect agricultural and industrial water use through changes in food demand and industrial structure [40, 41], thereby constituting a residual source of uncertainty in our projections. Therefore, using population size alone as a proxy for changes in total human water demand may not adequately capture the sector‐specific dynamics of water use, comparing with using the climate change [42, 43] and industrial growth to denote water use in the agriculture and industry sectors. Additionally, owing to the lack of data, our assumption did not consider the effects of infrastructure‐related water scarcity [36, 44] or water pollution [45], which likely led to an overestimation. Thus, the amount of feasible usable water is probably lower than we estimated. This also highlights the need to optimize water resource management, reduce water consumption, and mitigate water constraints on afforestation potential.

## 4 Experimental Section

### 4.1 Effects of Afforestation on Carbon Sinks

We used the Biome‐BGCMuSo model to assess the effects of afforestation on carbon sinks. Biome‐BGCMuSo is a process‐based model approach that simulates ecosystem carbon dynamics and cycles on the basis of climate, soil, and vegetation parameters [46, 47]. The Biome‐BGCMuSo simulates the storage and fluxes of water, carbon, and nitrogen between ecosystems and the atmosphere [48]. This model can simulate photosynthesis, respiration, allocation of organic matter, litter and decomposition of plant tissues, and circulation and migration of nutrients in different ecosystems [49]. The information of the driving data was described in Table S1. The key parameters of Biome‐BGCMuSo were calibrated in prior studies of China (Table S2) [11, 50]. Meanwhile, we compared our simulated NEP with flux site observation records obtained from the China Flux Observation and Research Network (http://www.chinaflux.org). Explicit information of these flux sites is described in Table S3. Notably, a high degree of consistency (R^2^≥0.52) among these comparisons indicates the reliability of our results (Figure S13). This approach captures the physiological and biogeochemical processes underlying carbon accumulation. The change in NEP between post‐afforestation and pre‐afforestation stages was used as a surrogate to denote the carbon sink contributed by afforestation on the basis of the pathway that we established in our prior studies [50, 51] and Text S1. Moreover, we considered the effects of future climate change on carbon sinks by using SSP‐245 and SSP‐585 climate data obtained from the CMIP6.

### 4.2 Identifying the Extent of Potential Afforestation Under Land Resource Constraints

First, we identified the afforestation potential under land resource constraints. Second, we quantified the effect of human water demand on afforestation. Finally, we measured the feasible afforestation potential under both constraints of land resources and human water demand. A flowchart illustrating the procedure is provided in Figure S14.

#### 4.2.1 Predicting the Potential Tree Cover and Forest Extent

We created massive sample points in nature reserves (regions with limited human activity) through random sampling (Figure S15). Then, we extracted the tree cover (from the CATCD tree cover product [52]) and environmental variables (5 climate, 2 topographic and 3 soil variables) at these sample points to fit a random forest regression model for predicting potential tree cover (Figure S16). The data sources for these environmental variables and tree cover are described in Table S4. Next, we used the trained model to project the potential tree cover across all of China. Finally, we converted the potential tree cover map into a potential forestland map (defined as >20% tree cover) (Figure S17).

#### 4.2.2 Quantifying the Extent and Area of Afforestation

Not all areas suitable for forest growth can be considered available for afforestation. We therefore excluded existing forestland, cropland on slopes <25°, wetland, and impervious surfaces to delineate the potential extent of forest restoration (Figures S2 and S18). To robustly identify the spatial distribution of these land use/cover (LUC) types, we harmonized five high‐resolution LUC products, including CLCD, ESA_WorldCover10, GLC_FCS30, GLAD and Esri Sentinel‐2 10m Annual Land Cover (Table S5). A majority voting approach was applied at the pixel level, whereby a given LUC class was assigned only when ≥3 products agreed on the classification (Figure S19). This strategy helps reduce classification uncertainty and enhances the reliability of the derived afforestation extent.

### 4.3 Afforestation Potential Under Constraints of Land Resource and Human Water Demand

To quantify the effect of water resource constraints on afforestation, we calculated the water resource supply and water resource demand at the basin scale. Then, we compared the magnitude of the water resource supply and demand in each basin. When the magnitude of the water supply is greater than the water demand, it implies that afforestation is feasible in this basin. In contrast, if the water supply is less than or equal to the demand, it implies that afforestation is not suitable in this basin. The explicit procedures and formulas used to quantify this effect and estimate the feasible afforestation potential are described in the following sections.

#### 4.3.1 Water Resource Supply

The magnitude of the water resource supply was calculated based on (Equation 1).

(1) $$WP_{i}=\left(PRE_{i}-\left(ET_{i}-ETA_{bw,i}\right)\right)\;\times A_{i}$$

where *WP_i_* denotes the magnitude of water resource supply in basin *i* (billion m^3^); *PRE_i_* denotes the magnitude of precipitation in basin *i* (mm); and *ET_i_* denotes the magnitude of water exhausted from ecosystems in basin *i* (mm), which is calculated from the Biome‐BGCMuSo model; *ETA*_*bw*,*i*_ denotes the blue water evapotranspiration in agriculture derived from irrigation in basin *i* (mm), which was calculated based on rule defined in Text S2; *A_i_* denotes the area in basin *i* (Mha). The cropland evapotranspiration (ET) derived from the Biome‐BGCMuSo model accounts for only the effects of precipitation and does not include irrigation. Water consumption from irrigation is calculated as water consumption and is therefore not considered here.

#### 4.3.2 Human Water Demand

The magnitude of human water demand was calculated with (Equation 2).

(2) $$WD_{i}=WDI_{i}+WDA_{i}+WDD_{i}$$

where *WD_i_* denotes the human water demand in basin *i* (billion m^3^); *WDI_i_* denotes the industrial water use in basin *i* (billion m^3^); *WDA_i_* denotes the agricultural water use in basin *i* (billion m^3^); and *WDD_i_* denotes the domestic water use in basin *i* (billion m^3^).

#### 4.3.3 Water Supply–Demand Balance

The water supply–demand balance was calculated based on (Equation 3).

(3) $$W\;S_{i}=WP_{i}\times \delta -WD_{i}$$

where *WS_i_* denotes the result from the water supply minus the water demand in basin *i* (billion m^3^); negative values indicate water deficit, where the water supply cannot meet the needs of humans, whereas positive values denote water surplus, such that the water supply can meet the needs of humans; and δ denotes the water resource utilization rate (%) in basin *i*.

To quantify the magnitude of water surplus available to support the afforestation area, we analysed the water supply‐demand balance in different basins and screened the basins where water surplus occurred. The magnitude of afforestation potential was calculated based on (Equation 4).

(4) $$WFA_{i}=WS_{i}/\Delta ET_{i}$$

where *WFA_i_* denotes the afforestation area that can be supported by the water surplus in basin *i* (Mha); Δ*ET_i_* denotes the change in water consumption between post‐afforestation and pre‐afforestation (mm), which is calculated from the Biome‐BGCMuSo model. Explicit information to estimate Δ*ET_i_* is described in Text S3.

#### 4.3.4 Afforestation Potential Under Constraints From Land Resource and Human Water Demand

The feasible afforestation potential under constraints from land resources and human water demand was calculated by (Equation 5).

(5) $$PFA_{t}=\min\left(LFA_{t},WFA_{t}\right)$$

where *PFA_t_* denotes the potential afforestation area under the constraints of land resources and human water demand in basin *t* (Mha); *LFA_t_* denotes the potential afforestation area under the constraint of land resources in basin *t* (Mha); and *WFA_t_* denotes the potential afforestation area under the constraint of the human water demand in basin *t* (Mha).

Then, we established (Equation 6) to identify the density of tree planting in each grid cell through compared the value of *LFA_t_* and *WFA_t_*

(6) $$DEN_{AF,t}=\left\{\begin{array}{c} 1,\;WFA_{t}\geq LFA_{t}\\ \frac{WFA_{t}}{LFA_{t}},\;WFA_{t}<LFA_{t} \end{array}\right.\;$$

where *DEN*_*AF*,*t*_ denote the density of tree planting in basin *t*. If *WFA_t_* ≥ *LFA_t_*, we defined *DEN*_*AF*,*t*_ as 1, indicating that human water demand does not further constrain afforestation in basin *t*, Thus, all grid cells suitable for afforestation under land‐resource constraints were afforested. If *WFA_t_* < *LFA_t_*, human water demand becomes the binding constraint, and *DEN*_*AF*,*t*_ represents the proportion of the land‐resource‐constrained afforestation potential that can be realized in each suitable grid cell.

The grid‐level feasible afforestation potential was obtained by multiplying the land‐resource‐constrained afforestation potential in each grid cell by *DEN_AF_* in all basins.

### 4.4 Scenario Description

#### 4.4.1 Scenario Setting Rules

To separate the effects of the constraints of land and water and the optimized management of land and water on China's afforestation potential and its carbon sink. We constructed 6 scenarios to address this issue. The explicit scenario setting rules and the meanings of these scenarios are described in Table 1. Each scenario contains three sub‐scenarios that consider climate change or not (the current climate state, SSP‐245 and SSP‐585) (Table S6). The motivation and assumptions underlying these scenarios are as follows:

**TABLE 1:** Summary of experimental simulation protocols.

Scenarios	Land resource constraints	Human water demand	Land optimizing	Water optimizing
Reference (R1)	—	—	—	—
Land Constraints (LCS)	°	×	×	×
Land and Water Resource Constraints (LWCS)	°	°	×	×
Land Management Optimization (LMO)	°	°	°	×
Water Management Optimization (WMO)	°	°	×	°
Land & Water Management Optimization (LWMO)	°	°	°	°

*Note*: ° indicates that the constraint is considered; × indicates that the constraint is ignored;—indicates no change from the current state.

**(1) Reference scenario (R1)**. The Reference scenario assumes that the forestland area remains constant during 2020–2060; this scenario denotes a reference used for comparison with policy‐based scenarios rather than a literal assumption.

**(2) Land constraint scenario (LCS)**. This scenario considers only the constraints of land resources on afforestation potential. In this scenario, we assume that trees will be planted in all areas suitable for afforestation except for current forestland, cropland, urban and wetland areas. With respect to the afforestation rate, we tentatively assume that the future afforestation rate will remain constant with the historical rate [11].

**(3) Land and water resource constraints scenario (LWCS)**. This scenario considers not only the constraints of land resources but also the constraints of human water demand. The water resource constraints imply that local water has to meet not only the needs of vegetation growth but also the needs of human water demand for agriculture, industry and domestic use.

**(4) Land management optimizing scenario (LMO)**. Considering the fact that cropland was the major land sources for afforestation in China during past decades [11, 53]. However, direct cropland conversion is now strongly constrained by China's food‐security strategy and the permanent basic cropland protection policy [54, 55]. In biophysically suitable afforestation areas, land may be occupied by cropland, wetlands, or built‐up land (Figure S2). Among these land‐use types, wetlands and built‐up land are generally unsuitable for improving land‐use efficiency through management‐based optimization because of their ecological protection requirements and fixed infrastructure functions. While China's cropland is highly fragmented and has relatively low land‐use efficiency [56], it has considerable potential for improving efficiency through optimized land management. This scenario assumes the implementation of optimized land management to increase the area of China's high‐quality cropland. The newly added cropland would then serve as a replacement for low‐productivity sloping cropland that is unsuitable for cultivation. It allows more marginal sloping cropland to be converted into forestland through afforestation programs under the condition that the overall cropland area is kept constant to guarantee food safety. This measure could effectively relieve the land resource constraint on afforestation. Land management optimization encompasses three pathways: the transfer of land‐use rights, land consolidation and the reclamation of abandoned cropland.

① **Transfer of land‐use rights (TLUR)**. This measure assumes that small and fragmented cropland are consolidated into large‐scale farmlands to eliminate field ridges and that all fragmented croplands with slopes <6° and parcel sizes ≤0.5 ha are integrated into large‐scale farmlands. The additional cropland area gained by removing field ridges can be calculated using (Equation 7).
(7) $$Area_{TLUR}=Area_{cropland\_f}\;\times cof_{ridge}$$

where *Area_TLUR_* denotes the increased cropland from the TLUR; $Area_{cropland\_f}$ denotes the fragmented cropland area with a slope less than 6°; and *cof_ridge_* denotes the proportion of field ridges to the total cropland area, which is obtained from published articles (Figure S20).

**② Land consolidation (LC)**. This measure assumes that China's rural population decreases substantially by 2060 with ongoing economic growth and accelerated urbanization. Thus, many rural residential buildings will be left vacant and uninhabited, and these vacant rural residential buildings have the potential to be reclaimed as cropland. The magnitude of additional cropland area gained by land consolidation is calculated by (Equation 8).
(8) $$Area_{LC}=\left(POP\_R_{2060}-POP\_R_{2020}\right)\;\times RPR$$

where *Area_LC_* denotes the increase in cropland from land consolidation and $POP\_R_{2060}$ and $POP\_R_{2020}$ denote the rural population in 2060 and 2020 (Figures S11 and S21), respectively, which are derived from the published article [57]. *RPR* denotes the per capita residential built‐up area in rural areas, which is obtained from the China Urban‒Rural Construction Statistical Yearbook [58].

③ **Reclamation of abandoned cropland (RAC)**. This scenario assumes that the abandoned cropland with a slope ≤6°is reclaimed. The magnitude of the additional cropland area gained by the reclamation of abandoned cropland is calculated by (Equation 9).
(9) $$Area_{RAC}=Area_{aba\_c}\;\times cof_{s6}$$

where *Area_RAC_* denotes the increase in cropland from the reclamation of abandoned cropland (Mha); $Area_{aba\_c}$ denotes the area of abandoned cropland, which is obtained from a published dataset (Mha) [59]; and *cof*_*s*6_ denotes the area ratio of abandoned cropland with a slope less than 6°.

(5) **Water management optimization scenario (WMO)**. In this scenario, sector‐specific water‐saving measures were implemented in addition to the baseline projections. Agricultural water use was reduced by improving irrigation water‐use efficiency in line with national targets, industrial water use was reduced by decreasing water‐use intensity, while domestic water use was reduced through lower leakage rates in public water‐supply networks. The water‐saving potential was quantified as the difference between baseline and optimized water use across the agricultural, industrial, and domestic sectors. Detailed assumptions and equations are provided in Text S4.

This scenario assumed that human water demand decreases when water‐saving strategies are implemented in the agricultural, industrial and domestic sectors. A decrease in human water demand would increase the water surplus to release the water constraint imposed on afforestation potential. The explicit measurement of the water savings potential in different sectors is described in Text S4.

**(6) Land and water management optimization scenario (LWMO)**. This scenario assumed land management optimization and water‐saving strategies are implemented together to relieve the constraints of land resources and water demand on afforestation potential.

#### 4.4.2 Scenario Comparison to Disentangle the Impact of Different Scenarios on Carbon Sink

To disentangle the effects of land and water constraints, as well as optimized land and water management, on afforestation‐induced carbon sinks, each scenario was compared with the reference scenario (R1). The explicit procedures were described in (Equation 10).

(10) $$\Delta CSP_{AF,S}=CSP_{AF,S}\;-CSP_{AF,R1}$$

where Δ*CSP*_*AF*,*S*_ denote the carbon sink potential change (g C m^−2^ year^−1^) between scenario S (including LCS, LWCS, LMO, WMO, LWMO scenario); *CSP*_*AF*,*S*_ denote the carbon sink potential (g C m^−2^ year^−1^) from afforestation in scenario S; *CSP*_*AF*,*R*1_ denote the carbon sink potential from afforestation (g C m^−2^ year^−1^) in scenario R1.

## Author Contributions

B.H.: conceptualization, methodology, software, investigation, writing – original draft, writing – review and editing, visualization, funding acquisition. F.L.: writing – review and editing, visualization, funding acquisition. Y.S.: writing – review and editing. X.W.: writing – review and editing. H.Z.: writing – review and editing. Z.O.: writing – review and editing, supervision.

## Conflicts of Interest

The authors declare no conflicts of interest.

## Supporting information




**Supporting File**: advs77990‐sup‐0001‐SuppMat.docx.

## Data Availability

The data that supports the findings of this study are available in Text S5 of this article. Any additional information required to reanalyze the data reported and Source data for graphs and charts in this paper is available from the lead contact upon request.
