# Supplementary material for: Optimized Land–Water Management Expands Afforestation Potential Under Land and Human Water‐Use Constraints
Source: Adv Sci (Weinh). 2026 Oct 11:e77990. Online ahead of print. doi: 10.1002/advs.77990 (PMC13655261; doi:10.1002/advs.77990)
Supplement: Supplementary file 1 [file ADVS-9999-e77990-s001.docx]

# Supporting Information for

Optimized land–water management expands afforestation potential under land and human water-use constraints

Binbin Huang^a, b^, Fei Lu^a, b, c*^, Yuebo Su ^d^, Xuming Wang ^e^, Hua Zheng^a, b^, Zhiyun Ouyang^a, b^

^a^ State Key Laboratory of Urban and Regional Ecology, Research Center for Eco-Environmental Sciences, Chinese Academy of Sciences, Beijing, 100085, China

^b^ University of Chinese Academy of Sciences, Beijing 100049, China

^c^ Beijing-Tianjin-Hebei Urban Megaregion National Observation and Research Station for Eco-Environmental Change, Beijing, 100085, China

^d^ Yunnan Key Laboratory of Forest Ecosystem Stability and Global Change, Xishuangbanna Tropical Botanical Garden, Chinese Academy of Sciences, Kunming, Yunnan 666303, China

^e^ Key Laboratory for Humid Subtropical Eco‐Geographical Processes of the Ministry of Education, Fujian Normal University, College of Geographical Sciences, Fuzhou 350007, China

# Contact Information:

^*^ Corresponding author: Fei Lu

Email: [feilu@rcees.ac.cn](mailto:feilu@rcees.ac.cn)

Tel: 86-010-62849815-4

Fax: 86-010-62849815

**This PDF file includes:**

- Text S1-S5
- Table S1 to S6
- Figures S1 to S24
- References

## Text S1. Estimation of carbon sink potential from afforestation using the Biome-BGCMuSo model

The carbon sequestration potential of afforestation was estimated using the process-based Biome-BGCMuSo model. Biome-BGCMuSo is a terrestrial ecosystem process model that simulates the coupled cycling of carbon, water, and nitrogen among vegetation, soil, and the atmosphere. The model explicitly represents major carbon fluxes, including gross primary productivity (GPP), autotrophic respiration (Ra), heterotrophic respiration (Rh), net primary productivity (NPP), and net ecosystem productivity (NEP). The model has been widely used to quantify the effects of ecological restoration and land-use/cover change on terrestrial carbon sinks in China. Here we estimate the carbon sink from afforestation through comparing the carbon sink change before and after afforestation based on the rules described as Equation S1.

${CSP}_{AF,i}={NEP}_{post,i}^{AF}-{NEP}_{pre,i}^{AF}$	(S1)

where ${CSP}_{AF,i}$ denotes the carbon sink potential from afforestation in grid *i* (g C m^-2^ yr^-1^); ${NEP}_{post,i}^{AF}$ denotes the NEP in grid *i* after afforestation (g C m^-2^ yr^-1^); ${NEP}_{pre,i}^{AF}$ denotes the NEP in grid *i* before afforestation (g C m^-2^ yr^-1^). Details to calculate the NEP in grid *i* was calculated in equation S2.

${NEP}_{i}=f_{F,i}\times{NEP}_{F,i}+f_{C,i}\times{NEP}_{C,i}+\cdots+f_{G,i}\times{NEP}_{G,i}$	(S2)

where ${NEP}_{i}$ denotes the NEP (g C m^-2^ yr^-1^) in grid cell *i*; $f_{F,i}$, $f_{C,i}$, and $f_{G,i}$ denote the area fraction of forest, cropland and grassland in grid cell *i*, respectively; and ${NEP}_{F,i}$, ${NEP}_{C,i}$, and ${NEP}_{G,i}$ denote unit-area NEP (g C m^-2^ yr^-1^) of forest, cropland and grassland in grid cell *i*, respectively.

## Text S2: Estimation of effective irrigation water and agricultural blue-water evapotranspiration

The agriculture water needs not only include green water (from precipitation), but also include blue water (from irrigation). If the green water supply could not feed the need of agriculture, more blue water is needed. The magnitude of blue water is not only affected by local climate background, but also affected by climate change. The detailed procedures and steps are described as follows:

The effective irrigation water was calculated based on the Equation S3.

$W_{a\_irr}=\varphi\times W_{irr}$	(S3)

where $W_{a\_irr}$ is the effective irrigation water that absorbed by crops; $\varphi$ is the irrigation water use efficiency coefficient, which obtained China’s water resources statistical yearbook; $W_{irr}$ is the total irrigation water withdrawal, which also obtained from China’s water resources statistical yearbook.

The contribution rate of effective irrigation water to blue water evapotranspiration was calculated based on the Equation S4.

${cof}_{bw}=\frac{{ETA}_{bw}}{W_{a\_irr}}$	(S4)

where ${cof}_{bw}$ is the contribution coefficient of effective irrigation water to agricultural blue-water evapotranspiration; ${ETA}_{bw}$ is the blue water evapotranspiration in agriculture derived from irrigation, which was calculated based on Equation S5.

${ETA}_{bw}=R_{maize}\times{ETA}_{bw\_maize}+R_{rice}\times{ETA}_{bw\_rice}+R_{wheat}\times{ETA}_{bw\_wheat}$	(S5)

where $R_{maize}$, $R_{rice}$, $R_{wheat}$ denotes the area ratio of maize, rice and wheat, respectively; ${ETA}_{bw\_maize}$, ${ETA}_{bw\_rice}$ and ${ETA}_{bw\_wheat}$ denotes the blue water evapotranspiration of maize, rice and wheat, respectively. The magnitude of blue evapotranspiration in these crops was described on Equation S6-S8.

${ETA}_{bw\_maize}={ETA}_{total\_maize}-{ETA}_{gw\_maize}$	(S6)

${ETA}_{bw\_rice}={ETA}_{total\_rice}-{ETA}_{gw\_rice}$	(S7)

${ETA}_{bw\_wheat}={ETA}_{total\_wheat}-{ETA}_{gw\_wheat}$	(S8)

where ${ETA}_{total\_maize}$, ${ETA}_{total\_rice}$, ${ETA}_{total\_wheat}$ denote the total evapotranspiration of maize, rice and wheat, respectively; ${ETA}_{gw\_maize}$, ${ETA}_{gw\_rice}$, ${ETA}_{gw\_wheat}$ denote the blue water evapotranspiration of maize, rice and wheat, respectively. We fitted six random forest model (Figure S22) through using the climate, soil and topographic variables as independent variables (Table S1), while the six variables as explanatory variables, respectively. The magnitude and which was obtained from the published article [1].

To quantify the effects of future climate change, the calibrated random forest models were driven by projected climate variables from the CMIP6 SSP2-4.5 and SSP5-8.5 scenarios. Soil and topographic variables were assumed to remain unchanged, whereas future climate variables were substituted for their historical counterparts. The predicted values under future climate scenarios were then used to estimate future blue-water evapotranspiration using Equation S6–S8.

## Text S3: Estimate evapotranspiration under the land/use cover change due to potential afforestation

Here, we estimated the afforestation caused the change of ET based on Biome-BGCMuSo model. First, we separated the China into 20×20 km^2^ grids (Figure S23). Then we calculate the ET change in each grid cell through calculating the effect of area ratio change in different land use/cover type in each grid before and after afforestation. The calculating of ET in each grid was described in Equation S9.

${ET}_{i}=f_{F,i}\times{ET}_{F,i}+f_{C,i}\times{ET}_{C,i}+\cdots+f_{G,i}\times{ET}_{G,i}$	(S9)

where ${ET}_{i}$ denotes the ET in grid cell *i*; $f_{F,i}$, $f_{C,i}$, and $f_{G,i}$ denote the area fractions of forest, cropland and grassland in grid cell *i*, respectively; and ${ET}_{F,i}$, ${ET}_{C,i}$, and ${ET}_{G,i}$ denote the area fractions of forest, cropland and grassland in grid cell *i*, respectively.

The effect of afforestation on ET change ($\Delta ET$) was described in Equation S10.

$\Delta{ET}_{i}={ET}_{post,i}-{ET}_{pre,i}$	(S10)

where $\Delta{ET}_{i}$ denotes the ET change in grid cell *i* after afforestation; ${ET}_{post,i}$, ${ET}_{pre,i}$ denote the ET in grid cell *i* after and before afforestation, respectively.

## Text S4: Projecting the future water use and estimate the water saving potential in agriculture, domestic and industry sectors

### (1) Agriculture sector

a) future water use

The water use in agriculture sector was mostly accomplished by the irrigation water. Here we tentatively assumed that the future cropland aera did not change. Thus, the future irrigation water was only affected by the climate change, which can be estimated based on the rules defined in following sections.

b) water saving potential

Current China’s irrigation water use efficiency coefficient is pretty lower than that in the developed countries, China’s government promise to continue improve the irrigation water use efficiency coefficient [2]. Thus, we tentatively assumed the water saving target made by government could be continued until 2060. The effect of water irrigation water use efficiency promotion on agriculture water use was calculated in equation S11：

$W_{irr\_opt,i}=\frac{W_{irr\_cur,i}\times{cof}_{irr_{cur,i}}}{{cof}_{irr\_cur,i}}$	(S11)

where the $W_{irr\_opt}$ denotes the irrigation water use after promoting irrigation water use efficiency; ${cof}_{irr\_opt}$ denotes the promoted irrigation water use efficiency; $W_{irr\_cur}$ denotes the current irrigation water consume (billion m^3^), which was obtained from the China water resources bulletin; ${cof}_{irr\_cur}$ denotes the current irrigation water use efficiency, which was obtained from the China water resources bulletin [3].

The magnitude of water saving potential in agriculture sector was calculated based on the equation S12:

$\Delta W_{irr\_sw,i}=W_{irr,i}-W_{irr\_opt,i}$	(S12)

where the $\Delta W_{ind\_sw,i}$ denotes the magnitude of water saving potential in agriculture sector (billion m^3^) in year *i*.

### (2) Industry sector

a) future water use

The water use in industry sector is not only affected by the industrial GDP, but also affected by the industrial water use intensity per unit of industrial value added. The future water use in industry sector was calculated based on the equation S13:

$W_{ind,i}={IWI}_{i}\times{GDP}_{ind,i}$	(S13)

where the $W_{ind,i}$ denotes the industrial water use in year *i*; ${IWI}_{i}$ denotes the industrial water use intensity per unit of industrial value added in year *i*; ${GDP}_{ind,i}$ denote the industrial GDP in year *i* that was obtained from the published GDP dataset [4].

b) water saving potential

China’s industry water intensity was much larger than developed countries, there is a great space to improve the industry water intensity [5]. According to that fact that China’s government long-term development plan, China would continue the improve the industry water intensity. Here we assumed China’s future industry water intensity would follow the historical rate, and reach the level of G7 developed countries until 2060 [5]. Meanwhile, we also considered the effect of future industry GDP growth on increasing industry water through combing the predicted GDP dataset (Figure S24) [4]. The effect of industry water intensity decreases on saving industry water can be calculated via Equation S14.

$W_{ind\_opt}={GDP}_{ind}\times{IWI}_{ind\_opt}$	(S14)

where the $W_{ind\_opt}$ denotes the intensity water use after promoting intensity water use efficiency in 2060; ${GDP}_{ind}$ denotes the industrial GDP; ${IWI}_{ind\_opt}$ denotes the industrial water intensity through implementing industrial water technology.

The magnitude of water saving potential in industrial sector was calculated based on the equation S15:

$\Delta W_{ind\_sw,i}=W_{ind,i}-W_{ind\_opt}$	(S15)

where the $\Delta W_{ind\_sw,i}$ denotes the magnitude of water saving potential in industrial sector (billion m^3^) in year *i*; $W_{ind,i}$ denotes the industry water use (billion m^3^) in year *i* ; $W_{ind\_opt}$ denotes the industry water use (billion m^3^) in year *i* through implementing industrial water technology.

### (3) Domestic sector

a) future water use

The domestic water use is not only affected by leakage rate of public water supply network, but also affected by the total population, and the Urban-rural population ratio. The magnitude of future domestic water was calculated based on Equation S16:

$W_{dom,i}=\frac{{POP}_{urban,i}\times{DWI}_{urban,i}+{POP}_{rural,i}\times{DWI}_{rural,i}}{(1-R_{leak})}$	(S16)

where the $W_{dom,i}$ denotes the domestic water use in year *i*; ${POP}_{urban,i}$ and ${POP}_{rural,i}$ denotes the urban and rural population in year *i*, which is calculated in Equation S17-S18; ${DWI}_{urban,i}$ and ${DWI}_{rural,i}$ denotes the urban domestic water use intensity and rural domestic water use intensity in year *i*, respectively. Their value was obtained from the water resource bulletin [3]; $R_{leak}$ denotes the leakage rate of public water supply network, which was obtained from China Urban–Rural Construction Statistical Yearbook [6].

To improve the consistency of projected population data with observed statistics, we calibrated the SSP-based projections of urban and rural populations in China using the corresponding values reported in the [7]. The specific calculation process is shown in Equation S17-S18:

$C{UPOP}_{urban,i}={UPOP}_{urban,2020}^{obs}\times\frac{{UPOP}_{urban,i}^{proj}}{{UPOP}_{urban,2020}^{proj}}$	(S17)

where $C{UPOP}_{urban,i}$ denotes the calibrated projected urban population in year $i$; ${UPOP}_{urban,2020}^{obs}$ denotes the observed urban population in year 2020 based on statistical yearbook data [7]; ${UPOP}_{urban,i}^{proj}$ and ${UPOP}_{urban,2020}^{proj}$ denotes the original SSP-based projected urban population obtained from published dataset [8] in year $i$ and 2020, respectively.

$C{RPOP}_{rural,i}={RPOP}_{rural,2020}^{obs}\times\frac{{RPOP}_{rural,i}^{proj}}{{RPOP}_{rural,2020}^{proj}}$	(S18)

where $CR{POP}_{rural,i}$ denotes the calibrated projected rural population in year $i$; ${UPOP}_{rural,2020}^{obs}$ denotes the observed rural population in year 2020 based on statistical yearbook data [7]; ${RPOP}_{rural,i}^{proj}$ and ${RPOP}_{rural,2020}^{proj}$ denotes the original SSP-based projected rural population derived from published data [8] in year $i$ and 2020, respectively.

b) water saving potential

Considering the fact that China’s leakage rate of public water supply network was pretty high than the developed countries. Meanwhile, the China’s government guaranteed to continue decrease the leakage rate of public water supply network. Thus, we tentative assumed the decrease rate of leakage rate of public water supply network would keep peace with historical rate. The effect of leakage rate of public water supply network decreases on domestic water can be calculated via Equation S19.

$W_{dom\_opt,i}=\frac{{POP}_{urban,i}\times{DWI}_{urban,i}+{POP}_{rural,i}\times{DWI}_{rural,i}}{(1-R_{leak\_opt,i)}}$	(S19)

where the $W_{dom,i}$ denotes the domestic water use in year *i*; ${POP}_{urban,i}$ denotes the urban population in year *i*; ${DWI}_{urban,i}$ denotes the urban domestic water use intensity in year *i*; ${POP}_{rural,i}$ denotes the rural population in year *i*; ${DWI}_{rural,i}$ denotes the rural domestic water use intensity in year *i*; $R_{leak\_opt}$ denotes the optimized leakage rate of public water supply network in year *i*.

The magnitude of water saving potential in domestic sector was calculated based on the equation S20:

$\Delta W_{dom\_sw,i}=W_{dom,i}-W_{dom\_opt,i}$	(S20)

where the $\Delta W_{dom\_sw,i}$ denotes the magnitude of water saving potential in domestic sector in year *i*.

### (4) All sector

The sum of all water management optimizing measures were calculated via equation S21:

$\Delta W_{sw,i}=\Delta W_{irr\_sw,i}+\Delta W_{ind\_sw,i}+W_{dom\_opt,i}$	(S21)

where the $\Delta W_{sw,i}$ denotes the water saving potential in year *i* after considering the effect of water management optimizing.

## Text S5: Data Availability Statement

The data are publicly available from the following resources. The WorldClim2.1 dataset is available at <https://www.worldclim.org/>. The Elevation data was produced by NASA with a spatial resolution of 90 m and can be downloaded from the USGS (<https://cgiarcsi.community/data/srtm-90m-digital-elevation-database-v4-1/>). The soil data was collected from the SoilGrids250m (<https://soilgrids.org/>). CLCD land use data was collected from Zenodo (<https://zenodo.org/records/8176941115>). ESA WorldCover 10 land use data was collected from <https://esa-worldcover.org/en>. GLC_FCS30 land use data was collected from <https://data.casearth.cn/thematic/glc_fcs30>. GLAD land use data was collected from <https://glad.umd.edu/dataset/GLCLUC2020>. Esri Sentinel-2 10m Annual Land Cover land use data was collected from <https://gee-community-catalog.org/projects/esrilc2020/>. Current climate data (2000–2020) were derived from station monitoring records in the China meteorological observation network and can be freely downloaded from the NOAA official website (https://www.ncei.noaa.gov/maps/daily/). Future predicted climate data derived from CMIP6 was obtained from the official website (https://esgf-data.dkrz.de/projects/cmip6-dkrz/CMIP6). CATCD tree cover was obtained from <https://zenodo.org/records/11047917>. The water resources data was obtained from the China Water Resources Bulletin (http://www.mwr.gov.cn/sj/tjgb/szygb/202510/t20251001_2067944.html). Any additional information required to reanalyse the data reported and Source data for graphs and charts in this paper is available from the lead contact upon request.

Table S1 Input datasets and parameters used to drive the Biome-BGCMuSo model.

| Data category | Variables | Temporal resolution | Data source |
| --- | --- | --- | --- |
| Meteorological forcing | Daily max/min temperature, precipitation, shortwave radiation, vapor pressure deficit (VPD) or relative humidity | Daily | Historical data: station monitoring records in the China meteorological observation network  Future data: CMIP6 global climate models |
| Atmospheric forcing | Atmospheric CO₂ concentration and nitrogen deposition | Annual | NASA and CMIP6 scenario datasets |
| Soil properties | Soil depth, layer thickness, sand, silt and clay fractions, bulk density | Static | SoilGrids |
| Land use/cover data | the area fraction of each land-cover type in each grid | Annual | Land-use datasets, forest inventories, and afforestation project records |

Table S2. Eco-physiological parameters of the Biome-BGCMuSo model.

| Parameters | EBF | ENF | DBF | DNF | Shrub | Grass | Cropland |
| --- | --- | --- | --- | --- | --- | --- | --- |
| C:N_leaf_ | 26[9] | 46[10] | 16.58[11] | 24.0[12] | 35[13] | 23.37[14] | 65[15] |
| C:N_lit_ | 65[9] | 93[10] | 49[11] | 49.0[12] | 75[13] | 45^[16]^ | 85[15] |
| C:N_fr_ | 99[9] | 90[10] | 43.2[11] | 42.0[12] | 58[13] | 23.37^[16]^ | 120[15] |
| C:N_lw_ | 82[9] | 50[10] | 50[11] | 50.0[12] | 50[13] | - | - |
| C:N_dw_ | 528[9] | 729[10] | 550[11] | 442.0[12] | 730[13] | - | - |
| *L*_lab_ | 0.32[9] | 0.32[10] | 0.20[11] | 0.39[12] | 0.3[13] | 0.29[14] | 0.68^[16]^ |
| L_cel_ | 0.44[9] | 0.44[10] | 0.51[11] | 0.44[12] | 0.45[13] | 0.55[14] | 0.23^[16]^ |
| FR_lab_ | 0.34[9] | 0.30[10] | 0.12[11] | 0.30[12] | 0.34[13], | 0.34^[16]^ | 0.34[15] |
| FR_cel_ | 0.45[17] | 0.45[10] | 0.54[11] | 0.45[12] | 0.44[13] | 0.44^[16]^ | 0.44[15] |
| DW_cel_ | 0.76[17] | 0.76[10] | 0.78[11] | 0.76[12] | 0.71[13] | 0.75^[16]^ | 0.75^[16]^ |
| W_int_ | 0.033[17] | 0.041[10] | 0.0355[11] | 0.041[12] | 0.045[13] | 0.027^[16]^ | 0.027^[16]^ |
| K | 0.58[17] | 0.5[10] | 0.599[11] | 0.5[12] | 0.55[13] | 0.60[14] | 0.48[15] |
| LAI_all:proj_ | 2[17] | 2.6[10] | 2[9] | 2.6[12] | 2.3[13] | 2.0^[16]^ | 2.0^[16]^ |
| SLA | 54.2[17] | 12.0[10] | 46.64[11] | 30.0[12] | 25.98[13] | 11.88[14] | 2.0^[16]^ |
| *SLA*_shd:sun_ | 2[17] | 2.0[10] | 2.0[11] | 2.0[12] | 2.0[13] | 2.0^[16]^ | 2.0^[16]^ |
| *G*_smax_ | 0.0065[17] | 0.0022[10] | 0.0094[11] | 0.005[12] | 0.006[13] | 0.006^[16]^ | 0.006^[16]^ |
| *G*_cut_ | 0.00001[17] | 0.00001[10], | 0.000094[11] | 0.00001[12] | 0.00006[13] | 0.00006^[16]^ | 0.00006^[16]^ |
| *G*_bl_ | 0.01[17] | 0.08[10] | 0.01[11] | 0.08[12] | 0.02[13] | 0.04[14] | 0.003[15] |
| *VPD*_s_ | 1100[17] | 930.0[10] | 1100[11] | 930.0[12] | 970[13] | 1000^[16]^ | 1000^[16]^ |
| *VPD*_c_ | 3600[17] | 4100.0[10] | 3600[11] | 4100.0[12] | 4100[13] | 5000^[16]^ | 5000^[16]^ |

Note: ENF: Evergreen needleleaf forest; DBF: Deciduous broadleaf forest; DNF: Deciduous needleleaf forest; C:N_leaf_: C:N of leaves; C:N_lit_: C:N of leaf litter; C:N_fr_: C:N of fine roots; C:N_lw_: C:N of live wood; C:N_dw_: C:N of dead wood; *L*_lab_ : Leaf litter labile proportion; L_cel_: Leaf litter cellulose proportion; FR_lab_: Fine root labile proportion; FR_cel_: Fine root cellulose proportion; DW_cel_: Dead wood cellulose proportion; W_int_ canopy water interception coefficient (LAI^−1^ day^−1^); k canopy light extinction coefficient; LAI_all:proj_ All-sided to projected leaf area ratio; *SLA* (projected area basis) (m^2^ kg^−1^ C); *SLA*_shd:sun_ Ratio of shaded SLA: sunlit SLA; *G*_smax_ maximum stomatal conductance; *G*_cut_ Cuticular conductance (m s^−1^); *G*_bl_ Boundary layer conductance (m s^−1^); *VPD*_s_: VPD: start of conductance reduction; *VPD*_c_: VPD: complete conductance reduction.

Table S3. Basic information on the available flux sites in China.

| Site name | Longitude | Latitude | Start-end time | PFT |
| --- | --- | --- | --- | --- |
| CBS | 128°05’45’’ E | 42°24’9’’ N | 2003-2010 | deciduous broadleaf forest |
| DHS | 112°32’3.8’’ E | 23°10’24’’ N | 2003-2010 | evergreen broadleaf forest |
| QYZ | 115°03’29.2’’ E | 26°44’29.1’’ N | 2003-2010 | evergreen broadleaf forest |
| XSBN | 101°15’55’’ E, | 21°55’39’’ N | 2003-2010 | evergreen broadleaf forest |
| HBG | 101°19’ E | 37°37’ N | 2003-2010 | shrubland |
| DX | 91°03’58.90’’ E | 30°29’50.21’’ N | 2004-2010 | shrubland |
| NMG | 116°24’14.4’’ E | 43°19’31.8’’ N | 2004-2010 | shrubland |

Table S4. CMIP6 global climate models, modeling institutions, and future climate scenarios used in this study.

| GCM | Modeling institution | SSP scenarios |
| --- | --- | --- |
| BCC-CSM2-MR | Beijing Climate Center, China | SSP-245, SSP-585 |
| CanESM5 | Canadian Centre for Climate Modelling and Analysis, Canada | SSP-245, SSP-585 |
| CNRM-CM6-1 | CNRM-CERFACS, France | SSP-245, SSP-585 |
| IPSL-CM6A-LR | Institut Pierre-Simon Laplace, France | SSP-245, SSP-585 |
| MIROC6 | MIROC, Japan | SSP-245, SSP-585 |
| MRI-ESM2-0 | Meteorological Research Institute, Japan | SSP-245, SSP-585 |
| NorESM2-MM | Norwegian Climate Centre, Norway | SSP-245, SSP-585 |
| GFDL-ESM4 | National Oceanic and Atmospheric Administration, Geophysical Fluid Dynamics Laboratory (NOAA-GFDL) | SSP-245, SSP-585 |

Table S5. Summary of environmental factors to establish the random forest machine learning regression model

| Data type | Variables | Spatial Resolution | Source |
| --- | --- | --- | --- |
| Climatic | Annual Mean Temperature | 30 seconds | WorldClim v2.1 (https://www.worldclim.org/) |
|  | Mean Temperature of Wettest Quarter | 30 seconds | WorldClim v2.1 (https://www.worldclim.org/) |
|  | Annual Precipitation | 30 seconds | WorldClim v2.1 (https://www.worldclim.org/) |
|  | Precipitation Seasonality | 30 seconds | WorldClim v2.1 (https://www.worldclim.org/) |
|  | Precipitation of Driest Quarter | 30 seconds | WorldClim v2.1 (https://www.worldclim.org/) |
| Topographic | Elevation | 90m | https://cgiarcsi.community/data/srtm-90m-digital-elevation-database-v4-1/ |
|  | Hillshade | 90m | https://cgiarcsi.community/data/srtm-90m-digital-elevation-database-v4-1/ |
| Soil | Soil Organic Carbon Stock | 250 m | SoilGrids250m (https://soilgrids.org/) |
|  | Sand content | 250 m | SoilGrids250m (https://soilgrids.org/) |
|  | Depth to Bedrock | 250 m | SoilGrids250m (https://soilgrids.org/) |

Table S6. Summary of land use/cover products used in this study

| Name | Spatial resolution (m) | Time | Sources |
| --- | --- | --- | --- |
| CLCD | 30 | 2020 | https://zenodo.org/records/8176941 |
| ESA WorldCover 10 | 10 | 2020 | https://esa-worldcover.org/en |
| GLC_FCS30 | 30 | 2020 | https://data.casearth.cn/thematic/glc_fcs30 |
| GLAD | 30 | 2020 | https://glad.umd.edu/dataset/GLCLUC2020 |
| Esri Sentinel-2 10m Annual Land Cover | 10 | 2020 | https://gee-community-catalog.org/projects/esrilc2020/ |

Figure S1. the water use duration in different sectors and scenarios. The water uses without or with considering water use saving technology in a) agriculture sector, b) industrial sector and c) domestic sector under SSP-245 and SSP-585 during 2020-2060; the water use change in 2060 VS 2020 d) without WMO (SSP-245); e) without WMO (SSP-585); f) with WMO (SSP-245); g) with WMO (SSP-585).

Figure S2. The spatial pattern of existing forest and potential afforestation opportunity under the constraint of land resources in China.

Figure S3. Spatial pattern of a) potential afforestation area and b) corresponding carbon sink under constraint of land resources and human water demand in 2060 (SSP-245); spatial pattern of c) potential afforestation area and d) corresponding carbon sink under constraint of land resources and human water demand in 2060 (SSP-585). $\boldsymbol{\Delta}\mathbf{NEP}$, change in net ecosystem productivity; N, northern China; NE, northeastern China; NW, northwestern China; S, southern China; SE, southeastern China; SW, southwestern China.

Figure S4. The spatial pattern of cropland increase supporting 1:1 afforestation from a) land transfer (LT); b) land consolidation (LC); c) abandoned land reclamation (RAC); d) land management optimizing (LMO).

Figure S5. Spatial pattern of a) potential afforestation area from LMO and b) corresponding carbon sink under constraint of land resources and human water demand in 2060 (SSP-245); spatial pattern of c) potential afforestation area from LMO and d) corresponding carbon sink under constraint of land resources and human water demand in 2060 (SSP-585). LMO: Land management optimization scenario.

Figure S6. The spatial pattern of water resource during a) current (2020); b) SSP-245 (2060); c) SSP-585 (2060).

**Figure S7.** The spatial patterns of human water demand and supply–demand balance under different water management strategies. The spatial pattern of human water demand under a) current (2020); b) SSP-245 without water saving strategies (2060); c) SSP-585 without water saving strategies (2060); d) SSP-245 with water saving strategies (2060); e) SSP-585 with water saving strategies (2060); and the spatial pattern of water supply–demand balance during f) current (2020); g) SSP-245 without water saving strategies (2060); h) SSP-585 without water saving strategies (2060); i) SSP-245 with water saving strategies (2060); j) SSP-585 with water saving strategies (2060).

Figure S8. Spatial pattern of a) potential afforestation area from WMO and b) corresponding carbon sink under constraint of land resources and human water demand in 2060 (SSP-245); spatial pattern of c) potential afforestation area from WMO and d) corresponding carbon sink under constraint of land resources and human water demand in 2060 (SSP-585). WMO: Water management optimization scenario.

**Figure S9.** The spatial pattern of dominant constraint factors for afforestation. The black area is the distribution of current forests.

Figure S10. The per capita domestic water use for urban and rural residents

Figure S11. China’s population dynamics during 2020-2060 (SSP-245/SSP-585). a) total population; b) urban population; c) rural population.

Figure S12. The magnitude and spatial patterns of water-supported afforestation potential under different climate and water management scenarios. a) Afforestation area supported by water resources surplus under different scenarios; the spatial pattern of afforestation area supported by water resources surplus in b) current (2020); c) 2060 without water saving management under SSP-245 scenario; d) 2060 without water saving management under SSP-585 scenario; e) 2060 water saving management under SSP-245 scenario; f) 2060 water saving management under SSP-585 scenario.

Figure S13. Comparison between the observed and simulated daily NEP based on Biome-BGCMuSo.

**Figure S14.** The flowchart for identifying afforestation potential under the constraints of land resources and human water demand.

Figure S15. The distribution of sample points and nature reserves in China.

Figure S16. Performance of the random forest model in predicting tree cover compared to the CATCD tree cover product: (a) training stage and (b) validation stage.

Figure S17. The spatial pattern of potential forestland in China.

Figure S18. The afforestation potential under the constrain of land resource in basin scale.

Figure S19. The spatial distribution of (a) existing cropland-forest-urban-wetland and (b) cropland distribution across slope classes in China

Figure S20. Field ridge coefficient in different subregions through literature search.

Figure S21. Spatial pattern of China’s population trend during 2020-2060 (SSP-245/SSP-585). a) urban population trend in SSP-245; b) urban population trend in SSP-585; c) rural population trend in SSP-245; d) rural population trend in SSP-585.

Figure S22. Comparison of Random Forest predicted ET versus gridded WFCP ET from Wang et al. (2023) for total ET and green ET across three major crops in China.

Figure S23. 20 km× 20 km grids that covered all of China.

Figure S24. The industry GDP change during 2020-2060 under SSP-245 and SSP-585 scenarios

## References

[1]	W. Wang, L. Zhuo, X. Ji, et al., “A gridded dataset of consumptive water footprints, evaporation, transpiration, and associated benchmarks related to crop production in China during 2000–2018,” *Earth Syst. Sci. Data* **2023**, *15* (11), 4803, <https://doi.org/10.5194/essd-15-4803-2023>.

[2]	H. Li, M. Li, Y. Wang, et al., “Precision irrigation framework could enhance water productivity and reduce carbon emissions in China,” *Communications Earth & Environment* **2025**, *7* (1), 113, <https://doi.org/10.1038/s43247-025-03137-9>.

[3]	Ministry of Water Resources of the People's Republic of China, “China Water Resources Bulletin 2020.” Ministry of Water Resources of the People’s Republic of China: Beijing, China, **2021**.

[4]	C. Jing, B. Su, J. Zhai, et al., “Gridded value-added of primary, secondary and tertiary industries in China under Shard Socioeconomic Pathways,” *Scientific Data* **2022**, *9* (1), 309, <https://doi.org/10.1038/s41597-022-01440-0>.

[5]	FAO, UN-Water, “Progress on change in water-use efficiency: Mid-term status of SDG Indicator 6.4.1 and acceleration needs, with special focus on food security and climate change.” Food and Agriculture Organization of the United Nations (FAO); UN-Water: **2024**.

[6]	Ministry of Housing and Urban-Rural Development of the People's Republic of China, “China Urban–Rural Construction Statistical Yearbook 2021.” Beijing, **2021**.

[7]	National Bureau of Statistics of China, “National Data,” <https://data.stats.gov.cn/dg/website/page.html#/pc/national/home>, accessed: 13 August 2026.

[8]	Y. Chen, X. Li, K. Huang, M. Luo, M. Gao, “High-Resolution Gridded Population Projections for China Under the Shared Socioeconomic Pathways,” *Earth's Future* **2020**, *8* (6), e2020EF001491, <https://doi.org/https://doi.org/10.1029/2020EF001491>.

[9]	H. Ren, L. Zhang, M. Yan, X. Tian, X. Zheng, “Sensitivity analysis of Biome-BGCMuSo for gross and net primary productivity of typical forests in China,” *Forest Ecosystems* **2022**, *9*, 100011, <https://doi.org/https://doi.org/10.1016/j.fecs.2022.100011>.

[10]	M. Yan, X. Tian, Z. Li, E. Chen, C. Li, W. Fan, “A long-term simulation of forest carbon fluxes over the Qilian Mountains,” *International Journal of Applied Earth Observation and Geoinformation* **2016**, *52*, 515, <https://doi.org/https://doi.org/10.1016/j.jag.2016.07.009>.

[11]	M. Kang, L. Zhu, H. Xu, T. Zha, Z. Zhang, “Modelling the responses of carbon and water fluxes with climate change for a poplar plantation in northern China based on the Biome-BGC model,” *Acta Ecologica Sinica* **2019**, *39* (7), 2378.

[12]	S. Li, L. Hou, A. Shi, L. Chen, X. Zhu, H. Bai, “Response of Larix chinensis forest ecosystem to climate change based on Biome-BGC model and tree rings,” *Acta Ecologica Sinica* **2018**, *38* (20), 7435.

[13]	Y. Zhang, M. Huang, J. Lian, “Spatial distributions of optimal plant coverage for the dominant tree and shrub species along a precipitation gradient on the central Loess Plateau,” *Agricultural and Forest Meteorology* **2015**, *206*, 69, <https://doi.org/https://doi.org/10.1016/j.agrformet.2015.03.001>.

[14]	L. Du, Y. Zeng, L. Ma, et al., “Effects of anthropogenic revegetation on the water and carbon cycles of a desert steppe ecosystem,” *Agricultural and Forest Meteorology* **2021**, *300*, 108339, <https://doi.org/https://doi.org/10.1016/j.agrformet.2021.108339>.

[15]	X. Xu, G. Yang, Y. Tan, et al., “Impacts of land use changes on net ecosystem production in the Taihu Lake Basin of China from 1985 to 2010,” *Journal of Geophysical Research: Biogeosciences* **2017**, *122* (3), 690, <https://doi.org/https://doi.org/10.1002/2016JG003444>.

[16]	M. A. White, P. E. Thornton, S. W. Running, R. R. Nemani, “Parameterization and Sensitivity Analysis of the BIOME–BGC Terrestrial Ecosystem Model: Net Primary Production Controls,” *Earth Interactions* **2000**, *4* (3), 1, <https://doi.org/10.1175/1087-3562(2000)004><0003:PASAOT>2.0.CO;2.

[17]	S. U. N. O. J. Li Xu-Hua, “Testing parameter sensitivities and uncertainty analysis of Biome-BGC model in simulating carbon and water fluxes in broadleaved-Korean pine forests,” *Chinese Journal of Plant Ecology* **2018**, *42* (12), 1131.
